# More Depression-Related Public Concern After the Suicide of a Pop Star in China: Evidence From the Online Big Data Platform

**DOI:** 10.3389/fpsyt.2021.629904

**Published:** 2021-04-06

**Authors:** Hong-Zhang Yu, Tian Fu, Jia-Nan Zhou, Ping Ke, Yun-Xia Wang

**Affiliations:** ^1^Faculty of Psychology, Naval Medical University, Shanghai, China; ^2^Third Xiangya Hospital of Central South University, Changsha, China; ^3^Air Force Hangzhou Special Service Recuperation Center Sanatorium Area 3, Hangzhou, China

**Keywords:** depression, public concern, mental health literacy, health information, internet

## Abstract

**Background:** In China, we have seen dramatic increases in public concern over depression and mental health after the suicide of some famous persons. The objective of this study is to investigate the changes of search-engine query patterns to monitor this phenomenon based on the tragic suicide of a young Chinese pop star, Kimi Qiao.

**Methods:** The daily search volume for depression was retrieved from both the Baidu Index (BDI) and the Sina MicroBlog Index (SMI). Besides, the daily BDI for suicide, schizophrenia, obsessive-compulsive disorder, common cold, stomach cancer, and liver cancer were collected for comparison. According to the time of Qiao's suicide, all data were divided into two periods (i.e., Period One from 1 September 2015 to 31 August 2016 while Period Two ranged from 1 October 2016 to 30 September 2017). The paired *t*-test was used to compare the differences in search volumes between two periods. The Pearson correlation analysis was used to estimate correlations between the BDI and SMI for depression.

**Results:** The average BDI for depression, BDI for suicide, and SMI for depression in Period Two were significantly higher than in Period One (*p* < 0.05). There was a strong positive correlation between the BDI and SMI for depression (*r* = 0.97, *p* < 0.001). And no significant difference in BDI for other diseases between the two periods was found.

**Conclusions:** The changes of search-engine query patterns indicated that the celebrity's suicide might be able to improve the netizens' concern about depression in China. The study suggests publishing more practical knowledge and advice on depression through the Internet and social media, to improve the public's mental health literacy and help people to cope with their depressive symptoms appropriately.

## Introduction

Qiao, a young Chinese pop star with a sunny and positive image, was found dead at home on 16 September 2016 in Shanghai, China. Almost at the same time, rumors of his death from “extreme sexual experiences” went viral on the Internet, nearly paralyzing the Sina MicroBlog (i.e., the most popular microblog in China). Later, the agent of Qiao released that Qiao was suffering from depression prior to his suicide (1). People frantically discussed depression on social media and searched for online information about depression. Moreover, we found a phenomenon from Chinese media data that the public seems to be very interested in issues involving celebrity depression. In some sense, the Chinese general public does begin to pay more attention to depression. To confirm this, we carried out this study.

According to the Depression and Other Common Mental Disorders Global Health Estimates by the World Health Organization, China has the highest number of people with depression in the world (2). More than 300 million people, or over four percent of the global population, were living with depression in 2015 (2). The core symptoms of depression are depressed mood, a loss of energy, anhedonia, irritability, difficulties in concentrating, feelings of worthlessness, and abnormalities in appetite and sleep (3). Depression is associated with reduced quality of life, decreased physical and social function, increased risk of suicide, and elevated personal and social-economic burden (4, 5). Therefore, it is of great importance to diagnose and treat depression early. Both psychological treatments and pharmacotherapy has been demonstrated to have satisfactory effects on treating depressive disorders (6, 7). However, not all people with depression seek professional treatments, especially those living in developing and undeveloped countries (8, 9). Barriers to treatment for depression include high treatment expenditure, cultural values, social stigma, and lack of knowledge about available treatments (10). All these lead to a significant number of individuals struggling with depression having not been discovered nor treated (11).

Likewise, the treatment of depression is not very optimistic in China. A recent report by Guo et al. (12) claims that tens of millions of depressive patients in China have posed a huge challenge to the Chinese mental health service system with limited public resources. Miller and Zhen find that only 10% of those experiencing depressive symptoms would seek professional medical services, while 90% choose to talk with their families and friends or do nothing (13). They also reveal that more than 70% of people do not know there is an institution for psychotherapy, and only half the people have little understanding of depression regardless of age, gender, and education. In fact, most depressive patients first go to the non-psychiatric section of general hospitals for help rather than to psychiatric hospitals in China (14–16). In addition, studies show that Chinese depressive patients are reluctant to talk about their mental symptoms to physicians because of traditional values and stigma, and the first things they often mention are the somatic symptoms (pain, gastrointestinal issues, weakness, and insomnia) (17–19). Fung et al. (20) also report that many Chinese people with mental illness do not seek help out of stigma and other unexplained reasons. Thus, it is urgent to promote the public's mental health literacy through proper channels (e.g., the Internet).

Nowadays, China has the broadest range of Internet users in the world. According to the 40th China Statistical Report on Internet Development, there were a total of 751 million netizens and 724 million mobile netizens as of 30 June 2017 (21). The report also claimed that people use the Internet for commerce transactions, finance, shopping, ordering meals, etc. Meanwhile, more and more people use the Internet to search for health-related information. In addition, the 2015 China Internet User Search Behavior Survey Report showed that 94.6% of the netizens used Baidu platform to search for information as of 31 December 2015. Baidu is the largest Chinese-language search engine and one of ten largest global websites. Baidu offers many services, including searching for websites, maps, videos, news, pictures, encyclopedia, multimedia files, translation, and other useful functions, as well as mobile service Baidu App (22).

Existing research has demonstrated that the availability of reliable health information for patients is of great importance; it can significantly increase self-efficacy, reduce anxiety, and enhance the self-care ability (23, 24). Notably, because of the low costs and rich resources, the Internet has become a major source for seeking health-related information, like providing options on how to access health services and professional advice (25, 26). Zhang et al. (27) find that 71.79% of Chinese adults have received health education on the Internet, and almost all (98.35%) had searched online for health-related information. They further find that Baidu is one of the most popular tools for seeking health-related information in China. Moreover, Chen and Zhu (28) argue that the Internet is an alternative source of help in times of mental illness, especially for those with internalized stigma. As for the reasons for this phenomenon, a few studies suggested that online health-related information is especially suitable for internet users because of the low cost and rich resources (29, 30). Thus, when confronted with depression, compared with other available sources, the Internet may be a more proper source of information for people with internalized stigma and limited access to health care services (31). Besides, all Internet traces would be recorded on big data platforms, which provide the possibility of utilizing those online data to carry out research.

In recent years, the big data platform has been used in a variety of novel studies on public health. For example, it has been found that there is a significant correlation between the number of people searching for influenza-related topics in Google and epidemiologic surveillance data on influenza outbreaks from the Centers for Disease Control and Prevention (32). The Chinese big data platforms have also been used widely in public health for issues including disease development trends, predictions of epidemics, internet surveillance, and the assessment of the impact of an emerging infectious disease (33–36). The present study employs the big data platform to study the netizens' concern about depression. To be specific, the study aims to: (1) compare the depression-related netizens' concern before and after the suicide of Qiao; and (2) explore depression-related search characteristics on the Internet.

## Materials and Methods

### Study Design

According to the time of the Qiao's suicide, the study was divided into two periods (i.e., Period One from 1 September 2015 to 31 August 2016 and Period Two from 1 October 2016 to 30 September 2017). Each period contains 12 months. September 2016 was excluded from the study to avoid the impact of extreme data, since the suicide occurred in that month and definitely aroused significant attention in China within a short time.

### Data Collection

The study used BDI (Baidu Index) to obtain information about age, gender, and geography. The age is divided into five groups (i.e., 10–19, 20–29, 30–39, 40–49, and above 50 years old). For the geographical features, this study selected the search volume T0P 10 cities (from 1 September 2015 to 30 September 2017). The study also used BDI to collect the Top 10 topics about depression from 1 September 2015 to 30 September 2017 on Baidu. Through these hot issues, we can learn what kind of information the netizens need.

To compare the netizens' concern about depression before and after the suicide of Qiao, this study collected the daily BDI for “抑郁症 depression” during two periods. The daily BDI for “自杀 suicide,” “精神分裂症 schizophrenia,” “强迫症 obsessive-compulsive disorder,” “感冒 common cold,” “胃癌 stomach cancer,” and “肝癌 liver cancer,” were also selected. For the parallel comparison, we count the daily SMI for “抑郁症 depression.”

### Research Instrument

Baidu Index is a systematic data-sharing platform based on Baidu search engine, which provides massive search behavior data (37). It provides its users with the search volume and trend for massive keywords and hot phrases, which can help to discover the changes of netizens' demands and monitor the trends of media netiznes' opinions. It calculates the “search volume index,” which represents the number of searches for keywords or hot phrases on the Baidu search engine. The bigger the BDI means the bigger the search volume. In addition, BDI also provides basic geographic (city, provinces, and regions where search queries are originated) and demographic (age, group, and gender) characteristics of users who submitted search queries related to a given search keyword. As such, the daily BDI is a measurement of the relative attention on a topic at a specific time point.

The Sina Micro Index (SMI) is another Chinese data-sharing platform based on the Sina MicroBlog. As the most popular micro blog in China, the Sina MicroBlog had a large number of active users, ~340 million as of 31 March 2017 (38). This platform can serve as a keyword research tool; on the basis of mass user behavior data and blog data, it can reflect the trends of netizens' opinions and keywords with weights on different topics.

### Statistical Analyses

The study launched SPSS 19.0 to conduct statistical analysis. The paired *t*-test was used to compare the differences in search volumes between two periods. The Pearson correlation analysis was used to estimate correlations between the BDI and SMI for depression. A *p* < 0.05 was considered statistically significant.

## Results

### Characteristics of Demographic and Geographic

Table 1 summarized the brief demographic descriptions of those who performed depression-related search queries on Baidu. The characteristics of user profiles were analyzed over two periods. From 1 September 2015 to 30 September 2017, the depression-related search queries were largely performed by people aged 20–49 (92%) and female (54%). The depression-BDI top 10 cities were Beijing, Shanghai, Guangzhou, Shenzhen, Hangzhou, Wuhan, Chengdu, Suzhou, Tianjin, and Nanjing, almost all of which are the GDP top ten cities in China. Results also showed an increase in the female who concerned about depression in the second period.

**Table 1:** Baidu depression-related searchers–demographic and geographic.

	**1st period**	**2nd period**	**1 September 2015–** **30 September 2017**
**Age**			
10–19	6%	6%	6%
20–29	24%	20%	20%
30–39	43%	47%	47%
40–49	25%	26%	25%
≥50	2%	1%	2%
**Gender**			
Male	51%	45%	46%
Female	49%	55%	54%
**Rank of city**			
1	Beijing	Beijing	Beijing
2	Shanghai	Shanghai	Shanghai
3	Guangzhou	Guangzhou	Guangzhou
4	Shenzhen	Shenzhen	Shenzhen
5	Wuhan	Hangzhou	Hangzhou
6	Tianjin	Chengdu	Chengdu
7	Hangzhou	Wuhan	Wuhan
8	Chengdu	Tianjin	Tianjin
9	Nanjing	Chongqing	Nanjing
10	Suzhou	Nanjing	Suzhou

### The Topics of Netizens Concern

Table 2 presented the top 10 depression-related searched topics on Baidu. Three kinds of topics mostly being concerned were summed up; the first one was “common knowledge of depression,” the second “treatment of depression,” and the third “diagnosis of depression.” It was also revealed that “common knowledge of depression” received the highest level of concern among the three topics.

**Table 2:** Top 10 depression-related topics on Baidu: 1 September 2015 to 30 September 2017.

**Rank**	**Depression-related topic**
1	What is depression?
2	Is the depression-test accurate?
3	How was Cui's depression cured?
4	Treatment for depression
5	How to treat depression?
6	Can depression be cured?
7	Self-treatment of depression
8	What does depression look like?
9	What are the symptoms of depression?
10	What medicine does depression take?

### Comparisons of BDI and SMI in Two Study Periods

**Figure 1:**
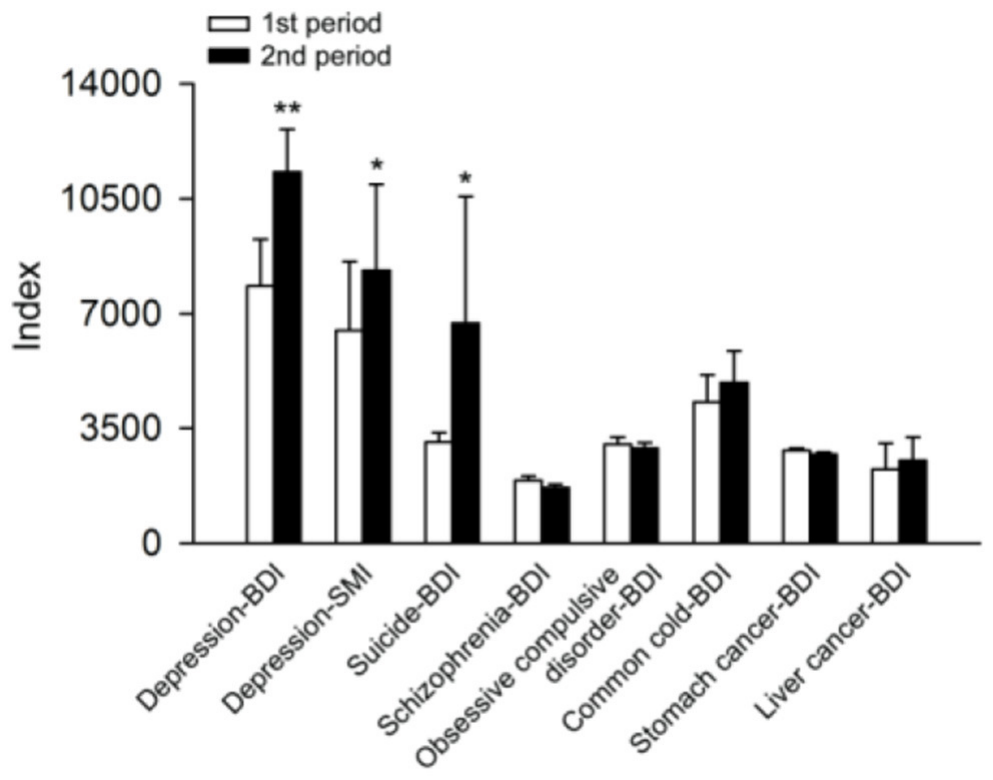
The comparison of average BDI and SMI in two study periods. *p < 0.05, **p < 0.01.

Figure 1 showed that the average BDI for depression, suicide-BDI and depression-SMI in Period Two were significantly higher than indexes in Period One (*P* < 0.05); the average BDI for schizophrenia in Period Two was significantly lower than the BDI in Period One (*P* < 0.05); additionally, there was no difference in Obsessive compulsive disorder-BDI, Common cold-BDI, Stomach cancer-BDI, and Liver cancer-BDI between two periods.

### The Trends of BDI and SMI

**Figure 2:**
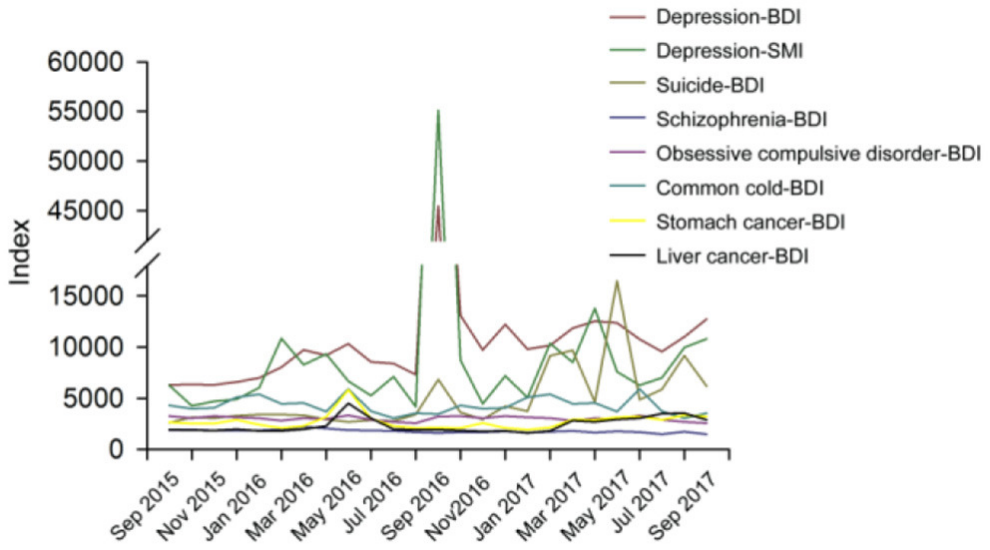
Index for Depression-BDI, depression-SMI, Suicide-BDI, Schizophrenia-BDI, Obsessive compulsive disorder-BDI, Common cold-BDI, Stomach cancer-BDI, Liver cancer-BDI: 2015.09-2017.09. Marked date 16 September 2016: the suicide time of Qiao, 17 September 2016: Qiao's agent announced that he had died by suicide because he could not afford to suffer from depression.

**Figure 3:**
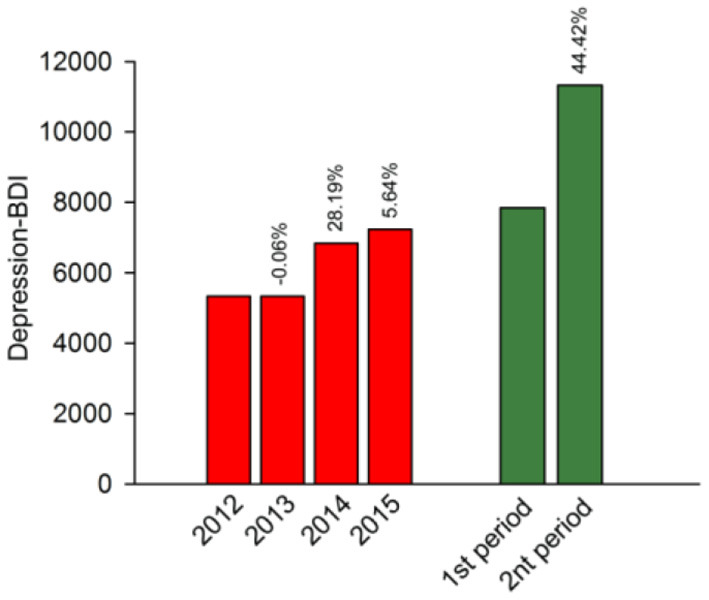
The yearly average depression-BDI from 2012 to 2015 and average depression-BDI in two study periods. The percentage represents the growth rate relative to the previous year or period.

The search interest for the Chinese term of “抑郁症 depression” in BDI and SMI has increased steadily since 1 September 2015 and closely reflected the term “自杀 suicide” in BDI. The depression-BDI rapidly increased from 6,571 on 15 September 2016 to its peak at 472,797 on 17 September 2016, declined to 15,730 with fluctuations on 30 September, and stayed above the primary level before 16 September. Similarly, the depression SMI reached a peak of about 775,845 on 17 September, and gradually declined to 14,309 on 30 September. The average BDI for depression was significantly positively correlated with the average SMI for depression (*r* = 0.97, *p* < 0.001). Furthermore, the BDI for schizophrenia, obsessive compulsive disorder, common cold, stomach cancer, and liver cancer in China were mapped in Figure 2, none of which show a positive trend. For the average BDI for depression, the annual rate of growth from 2012 to 2015 (11.26%) was significantly lower than the increase (44.42%) during the study time (see Figure 3).

## Discussion

The present study examined whether the Chinese netizens pay more attention to depression after the Qiao's suicide. The results confirmed that view, and the paper also attempts to explore depression-related search characteristics on the Internet (This study investigated the differences in search-engine query patterns before and after the Qiao's suicide to monitor the changes of netizens' concern about depression).

We find that the number of people focusing on depression in China is increased year by year, especially in recent years. On the one hand, we need to acknowledge that the growing popularity of mobile networks has brought convenience to the general netizens in China, prompting more people to search for information on the Internet. According to Statistics Bulletin of China Communications Operation in 2016, China has 5,590,000 mobile phone base stations, of which 2,630,000 are 4G base stations, and 777 million 4G users (39). These big data illustrated the potential that people use the Internet to seek health-related information. People can easily get much health-related information on the Internet.

On the other hand, we have to recognize the netizens' inner demand for mental health. With the rapid economic development of China in the last two decades, the Chinese living standard has been greatly improved. Meanwhile, it also brought a hurried lifestyle and more stress to the Chinese people (40). Numerous studies have considered stress to be a potential environmental risk factor for many psychological and behavioral abnormalities, including depressive disorders (41–43). Similarly, there are some clues in this research. The depression-BDI top 10 cities in China were Beijing, Shanghai, Guangzhou, Shenzhen, Hangzhou, Wuhan, Chengdu, Suzhou, Tianjin, and Nanjing, which were almost in line with the Chinese government's announcement of the top 10 GDP cities in 2015 (44). The study also found that the vast majority of Baidu searches for “depression” came from users aged 20–49 (92%). As known, people aged 20–49 are the main labor force in any country. This study thus offers some support for the relationship between stress and depression. Actually, previous studies had found that chronically persisting environmental and work-related stress could lead to depression (45, 46). However, some studies hold a different view that better quality of life promotes more netizens' awareness on mental health and more attention to depression (47–49). From the author's point of view, perhaps these two views are both at work.

In addition, the study showed that females were more concerned about depression than males. Although our results were unable to tell whether there were some important differences between males and females suffering depression, a few studies have reported that women were more likely to suffer from depression and exhibited more serious depressive symptoms than men (50, 51). A recent study reveals that women under high stress from both work and family are five times more likely to experience suicidal ideation (51). Nowadays, more Chinese women become irreplaceable in the occupational arena with a nearly 70% labor force participation rate (52). However, females are faced with higher work stress and conflict than males (53), they also had to bear the family stress and take traditional responsibilities such as household work and children's parenting (54). The family roles (daughter, wife, mother, sister, daughter-in-law, etc.) and the social roles (colleague, leader, etc.) exist simultaneously, resulting in more conflict. Therefore, when it comes to female depression, it is essential to regard work and family stress as the combined exposure. Therefore, we suggest paying more attention to female depressive patients, especially those working females.

Suicide is the main cause of death among Chinese people aged 15–34 years, while in the global population aged 15–29 years, it is the second leading cause of death (55, 56). Apart from depression, there were a series of other risk factors for suicide including low levels of education, heavy work fatigue, low income, staying single, poor health condition, et cetera. A recent study revealed that people with high depression and high anxiety were 54.77 times more at risk for suicide, much higher than people with either high anxiety or high depression (56). Increasing work pressure of young Chinese adults might take responsibility for this phenomenon. The low socio-economic status caused by unemployment can be one of the risk factors for suicide, but the exposure to various stressful events of those employees cannot be ignored either (57). Working characteristics such as occupation types and periods have been proved to result in different levels of work stress (58, 59). If people have irregular working schedules such as rotating shift work and excessive working time, they will feel highly stressed and perform poorly in work, especially for medical staff and police officers (60). Besides, it is found that suicide ideation is common among employed populations (prevalence range: 3.1% to 27.5%) and closely related to high work stress (61).

The increase in mobile phone users certainly can prompt more depression-related search volumes, but we should not ignore the major social events' impacts on the netizens' concern about depression. Table 2 involved Cui's topic, which has been discussed for more than a decade in China. Cui is a well-known TV media personality and the story of his recovery from depression has brought hope and inspiration to many depressive patients and their families and friends. This demonstrates the positive guiding role of the media in public health.

Our study found that there were significant differences in the average BDI for depression, SMI for depression, and BDI for suicide between two periods, but other diseases did not show such difference. This is consistent with existing empirical evidence that depression is significantly related to suicide (62, 63). All these results suggest that the suicide of Qiao had actually caused more concern among netizens about depression. This was verified by the growth rates of BDI in Figure 3. These two depression-related events have caused great concern among netizens about depression, leading us to think seriously about the reasons behind it. Cui's event made more people realize that depression can be cured, but it is more important to make people aware that depression is a kind of disease like the common cold, but not a shameful thing. The suicide of Qiao revealed the concealment of depression. A person who looks sunny and works seriously may be a depressive patient. A few researchers have mentioned that more and more people are struggling with depression nowadays, but it was difficult to perceive (64–66).

The study acquired depression-related topics that people are concerned about as well. The topic “common knowledge of depression” received the highest level of concern. This topic contains “what is depression” and “what are depressive symptoms,” meaning that many people lack common sense about depression in China. Previous studies have found that most people have low levels of mental health literacy in China, especially in remote rural areas (67, 68). In fact, this is a universal phenomenon in the developing world; Loh and Joshi (69) also claimed that Indians lack sufficient awareness of depression and are more likely to label their depressive symptoms as “tension” or “worry” (69). Limited mental health literacy often means poor treatment and lower recognition rates of mental illness, including depression (70, 71); in other words, better knowledge and more positive beliefs about depression are associated with positive response to treatment (72). Therefore, increasing people's awareness of depression should be given serious consideration, which can promote service utilization (73, 74).

In the past decade, psycho-education has been recognized as an effective way to provide information regarding psychological disorders and their treatments, encouraging patients and their families to actively cope with disorders, including depression (75, 76). The Chinese government and some health-related organizations have strengthened the education of common sense about depression in recent years. However, there is still much work that needs to be done, such as establishing professional organizations, training staff, improving education methods, and so on. The second topic people were concerned about was the treatment of depression, especially the therapeutic effects. A large number of people are not informed or are completely unaware of the therapeutic effects of depression. This may be because there are not enough resources to treat depression in China (77).

Actually, these problems not only exist in China, but also in other developing countries, even in the United States. A report conducted by Kataoka et al. (78) demonstrated that eighty percent of American youth cannot get access to mental health services. The third topic was the diagnosis of depression, especially the accuracy of the self-test scale. This means that a significant number of people want to measure or have measured whether they are depressive or not. From this, we infer that some people may prefer to self-test because of stigma. A previous report also shows a similar point of view that some Chinese depressive patients seek help from the Internet because of the stigma (79). This partly explains why there are so many depressive patients in China, and a lack of professional treatments.

Nowadays, more and more people search for information about depression on the Internet. This is a quite exciting phenomenon, which is of great help to the popularization of depression-related knowledge and the public improvement of national happiness. Intellectually, we need to recognize the fact that most people just know that they need to search for information about depression, but do not know how to identify what they really need and what is reliable. Not to mention those who are just attracted by advertising. Sadly, the Internet is riddled with unprofessional health-related information that misleads people to make wrong decisions and results in serious consequences eventually (80–82). A recent report by Zhang et al. (27) indicates that only fourteen percent of the Chinese believe that health-related information on the Internet is effective and professional. This situation presents us with a new challenge: How to convey professional health information to the netizens on the Internet or social media? It is a new problem that every country in the world needs to take into serious consideration. Our study provides a message about the role of celebrities in public health information transmission. Nevertheless, the publicity of celebrities committing suicide is a double-edged sword, which may be positive or negative. Consequently, we should report more positive aspects of depression-related reports to guide the netizens' understanding of depression.

Big data can give us clues that we have not been able to study before, but it still doesn't cover everything. During the study period, there were also news reports of government officials, university professors, college students, and others who died by suicide because of depression. While these events have led to netizens' increased awareness of depression, their impact on the study itself has been limited, as this paper focuses more on trend changes. Actually, besides some subject factors, there are possibly a series of objective factors such as seasons which also influence depression-related netizens' concern, which are quite complicated to study. Consequently, in order to concentrate more on our own theme, we only discuss the influence taken by social suicide incidents similar to Qiao's from the perspective of social psychology here, which we expect to provide reference to improve netizens' mental health.

While also considering the limitations of the study, we hope to be able to use more detailed data from the big data platform. This is beneficial to cross-sectional comparative studies and prospective studies. In the future, we also expect to use both online and offline resources to enrich our research.

## Limitations

Several limitations exist with the use of BDI to examine the netizens' health concern. Firstly, BDI only captures data from Internet users. There are a large number of non-internet users in China, especially those in remote areas and who do not like surfing the Internet. Thus, we just considered our study objects as netizens instead of all of the population in China. Secondly, the study only used the Chinese term of “抑郁症 depression” which might have limited access to data. Thirdly, although the study has described some basic information, there are still many discrepancies on search-engine query patterns for netizens with diverse sociodemographic backgrounds. For instance, the elderly populations are slower to adopt technology, including the internet. This factor cannot be ignored, since China is stepping into an aging society. Lastly, the search volume index was influenced by random factors, which is an unavoidable limitation.

## Conclusions

The changes of search-engine query patterns indicated that the celebrity's suicide might be able to improve the netizens' concern about depression in China. The study suggests publishing more practical knowledge and advice on depression through the Internet and social media, to improve the netizens' mental health literacy and help people to cope with their depressive symptoms appropriately.

## Data Availability Statement

The original contributions presented in the study are included in the article/supplementary material, further inquiries can be directed to the corresponding author/s.

## Author Contributions

H-ZY designed the study, analyzed the data, data interpretation, drafted the manuscript, and revised new drafts based on input from co-authors. TF participated in data interpretation and drafted the manuscript. J-NZ and PK participated in the design of the study and the statistical analysis. Y-XW participated in the design of the study, collected data, and contributed to drafting the manuscript. All authors contributed to the article and approved the submitted version.

## Conflict of Interest

The authors declare that the research was conducted in the absence of any commercial or financial relationships that could be construed as a potential conflict of interest.

## Abbreviations

BDI: Baidu Index
SMI: Sina MicroBlog Index.
